# Multi-Attack Intrusion Detection for In-Vehicle CAN-FD Messages

**DOI:** 10.3390/s24113461

**Published:** 2024-05-27

**Authors:** Fei Gao, Jinshuo Liu, Yingqi Liu, Zhenhai Gao, Rui Zhao

**Affiliations:** 1 State Key Laboratory of Automotive Simulation and Control, Jilin University, Changchun 130025, China; gaofei123284123@jlu.edu.cn; 2 College of Automotive Engineering, Jilin University, Changchun 130025, China; jsliu23@mails.jlu.edu.cn (J.L.); gaozh@jlu.edu.cn (Z.G.); 3 Strategic Cooperation Department, Mobje Co., Ltd., Changchun 130013, China; yingqi.liu.b-m@faw-vw.com

**Keywords:** CAN-FD, anomaly detection, LSTM, attention mechanism, deep learning, vehicle security

## Abstract

As an enhanced version of standard CAN, the Controller Area Network with Flexible Data (CAN-FD) rate is vulnerable to attacks due to its lack of information security measures. However, although anomaly detection is an effective method to prevent attacks, the accuracy of detection needs further improvement. In this paper, we propose a novel intrusion detection model for the CAN-FD bus, comprising two sub-models: Anomaly Data Detection Model (ADDM) for spotting anomalies and Anomaly Classification Detection Model (ACDM) for identifying and classifying anomaly types. ADDM employs Long Short-Term Memory (LSTM) layers to capture the long-range dependencies and temporal patterns within CAN-FD frame data, thus identifying frames that deviate from established norms. ACDM is enhanced with the attention mechanism that weights LSTM outputs, further improving the identification of sequence-based relationships and facilitating multi-attack classification. The method is evaluated on two datasets: a real-vehicle dataset including frames designed by us based on known attack patterns, and the CAN-FD Intrusion Dataset, developed by the Hacking and Countermeasure Research Lab. Our method offers broader applicability and more refined classification in anomaly detection. Compared with existing advanced LSTM-based and CNN-LSTM-based methods, our method exhibits superior performance in detection, achieving an improvement in accuracy of 1.44% and 1.01%, respectively.

## 1. Introduction

Intelligent connected vehicles are equipped with advanced sensors, enabling interaction with the external environment. They involve extensive data and code, providing functionalities for driving assistance and cockpit interaction. With the widespread adoption and increasing sophistication of communication interfaces, external attackers are able to intrude into the in-vehicle network and subsequently target the Electronic Control Units (ECUs) connected to this network. ECUs are critical components within autonomous vehicles, playing an essential role in processing real-time decisions and data exchange [1]. Securing communication among ECUs is pivotal in thwarting potential cyberattacks and safeguarding in-vehicle operational safety.

**Figure 1:**
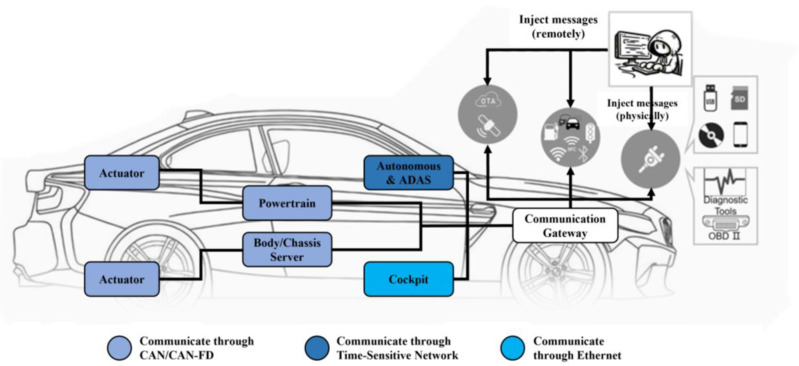
In-vehicle CAN/CAN-FD architecture and attack surface.

ECUs in vehicles widely use Controller Area Network (CAN)/Controller Area Network with Flexible Data (CAN-FD) Rate bus for communication. The original CAN/CAN-FD bus design does not adequately consider information security [2], lacking sender verification and message authentication in its broadcasting mechanism, which makes in-vehicle networks vulnerable to tampering and injection attacks. As depicted in Figure 1, the increasing connectivity of modern vehicles with external devices [3], such as through OBD-II ports [4], USB ports, Wi-Fi, and Bluetooth, exacerbates this vulnerability [5,6]. Once network attackers gain access to the bus [7], they can manipulate communication, including injecting anomalous frames that transmit various erroneous commands or data to targeted vehicle components. The prevalent types of abnormal messages include Denial of Service (DoS) [8], Fuzzing [9,10], Replay [11], Spoofing [12], Scaling attack [13,14], and Ramp attack [13,14]. Researchers have explored the security issues of in-vehicle networks [15]. Koscher et al. [11] are among the first to show how attackers gain complete control over vehicle functions by intercepting and reverse-engineering CAN bus data. This unauthorized access can disrupt critical automotive functions, like braking and steering, raising significant safety risks. It can also expose personal data such as vehicle movements and driving preferences. Through the analysis of the past ten years, it was found that in the most common automotive attack incidents, those directly or indirectly caused by the CAN/CAN-FD bus accounted for 13.61% of the total [16]. Therefore, enhancing the security of CAN/CAN-FD systems is essential to protecting in-vehicle networks from increasing cyber threats.

Intrusion Detection Systems (IDS) are crucial for securing in-vehicle networks by effectively identifying and mitigating cyberattacks on CAN/CAN-FD [17,18]. Intrusion detection algorithms are generally classified into three types: periodicity-based, semantic-based, and data-based [19], each varying in their detection principles and scope. WP29 R155 [20], ISO 21434 [21], and Cybersecurity Best Practices for the Safety of Modern Vehicles [22] all emphasize that anomaly detection is an effective and necessary means to prevent vehicle attacks.

Periodicity-based IDS [23,24,25] primarily focus on the temporal characteristics of network communication, particularly the periodicity of messages. These systems detect anomalies by analyzing deviations from expected transmission intervals. Halder et al. [23] proposed an IDS utilizing the clock offset of the transmitting ECU to monitor the in-vehicle network and identify anomalies using the Cumulative Sum method. Olufowobi et al. [24] developed an IDS based on real-time schedulable response time analysis of CAN buses, identifying anomalies by comparing the actual completion time of messages with the forecasts from a constructed message timing model. Ji et al. [25] employed clock drift to discern timing patterns in CAN bus messages for intrusion detection. However, these IDS can miss non-periodic or dynamically timed messages, which could allow irregular attack patterns to go undetected. Such limitations stem from the variable nature of some CAN/CAN-FD messages, and attackers might exploit this by mimicking normal message periodicity, thereby evading detection. Additionally, some attacks that do not alter message timing can remain undetected, reducing the effectiveness of periodicity-based IDS.

Semantic-based IDS [26,27,28] concentrates on the analysis of the meaning or logic of message contents to ensure alignment with system operations. The approaches verify that messages adhere to expected formats and values. Narayanan et al. [26] employed a hidden Markov model to identify abnormal states in CAN messages, capturing data via a sliding window and interpreting it as observation sequences. This model calculates probabilities to pinpoint abnormal signals. Guo et al. [27] implemented a ring architecture with multiple sensors, applying edge-based data fusion for anomaly detection. Wasicek et al. [28] introduced a context-aware intrusion detection method for anomalies in automotive control systems. Semantic-based IDS require a thorough understanding of CAN protocols and message behaviors, and system updates may necessitate reconfigurations. These systems might not detect new attack types that deviate from known patterns. Furthermore, semantic-based IDS is typically more complex than other types of IDS, as it needs to discern complex content, structure, and contextual meanings.

Data-domain-based IDS [19,29,30,31,32,33] utilizes machine learning and data analysis to identify network anomalies by analyzing extensive network traffic. These models are trained to recognize normal behavior patterns and detect deviations, adapting well to dynamic network environments. Wang et al. [29] introduced an IDS utilizing Hierarchical Temporal Memory learning. In their approach, a binary data stream is fed into each data sequence predictor prior to decoding, and the predicted output is then evaluated using an anomaly scoring mechanism. Based on prediction, Taylor et al. [30] developed a neural network with a Long Short-Term Memory (LSTM) [34] layer to predict the next package. The error measure is made using the predicted output value of the log loss function, and its error is used as a signal for detecting anomalies in the sequence. Based on classification, M. D. Hossain et al. [31] developed attack datasets for the CAN system and established an LSTM-based model for anomaly defense. Building on LSTM, Chen et al. [32] introduced causality into the method for anomaly detection that exhibited commendable performance. Also based on LSTM, Qin et al. [33] introduce an anomaly detection algorithm for the CAN bus system, aimed at reducing false positives and maximizing detection accuracy by evaluating a range of loss functions. However, challenges include difficulty in precisely identifying attack types and implementing specific countermeasures, and the scarcity of real-vehicle data for training robust models. These models often rely on simulated data, limiting their effectiveness in real-life scenarios. Furthermore, there is a continuous need for enhancement in the accuracy of these data-driven. False Negatives (FNs) and False Positives (FPs) remain a concern. Improving accuracy is crucial for the reliability of these systems in practical applications.

In this work, we propose a data-driven intrusion detection model comprising two sub-models: the Anomaly Data Detection Model (ADDM) and the Anomaly Classification Detection Model (ACDM). ADDM, based on LSTM, is designed to identify anomalous frames within CAN-FD datasets containing attacks, utilizing a stacked LSTM layer structure that deepens the neural network and allows each layer to further refine information learned from its predecessors. This methodology aids the model in effectively learning and capturing complex features and long-range dependencies inherent in time-series data. The ACDM model enhances stacked LSTM layers with temporal attention [35] to categorize anomaly types in CAN-FD frames. LSTM networks can learn the temporal features of repetitive request patterns observed in DoS and Replay attacks. The attention mechanism assists the model in focusing on the most crucial elements of the current predictive task, such as identifying tampered messages during Spoofing attacks. Furthermore, the attention mechanism dynamically adjusts the weights of input features, enabling the model to automatically adapt its focus on attack characteristics learned from historical data, such as the random or anomalous data patterns in Fuzzing attacks. Our approach surpasses the limitations of frequency-based detection that depends on periodic messaging. Grounded in extensive experiments with real CAN-FD frame data from actual vehicles, our method achieves high accuracy in identifying various types of anomalies without needing extensive prior knowledge, unlike semantic-based IDS.

The main contributions of this paper can be summarized as follows:

1. Our model employs LSTM layers with attention mechanisms to innovatively detect various CAN-FD bus attacks, such as DoS, Fuzzing, Replay, Spoofing, Scaling, and Ramp. This detection approach does not require the CAN-FD communication matrix or extensive prior knowledge, enhancing its broad applicability.
2. As far as we know, we are the first to collect raw CAN-FD bus message data directly from actual vehicles and generate an attack dataset based on it. This dataset reflects diverse attack patterns, offering high adaptability for simulating scenarios across various vehicular environments.
3. To validate our method’s effectiveness, we conduct experiments on two datasets: the real-vehicle dataset and the automotive CAN-FD Intrusion Dataset from the Hacking and Countermeasure Research Lab [36] in Korea. Compared with the State-of-the-Art (SOTA) method, our model provides refined classification capabilities for different types of attacks. Experimental results show that our model improves attack classification accuracy by 1.01% over the SOTA method, effectively identifying the type of attack in anomalous frames.

The rest of the paper is organized as follows: Section 2 introduces the background of this work, including the structure of the CAN-FD frame and types of attacks. Section 3 introduces the problem definition. Section 4 focuses on the research methods of this paper, presenting methodology encompassing data collection, generation of abnormal datasets, data preprocessing, and the architecture of two sub-models. Section 5 introduces the experimental results of this method and carries out comparisons and discussions. Section 6 concludes this work and highlights the scope for future work.

## 2. Background

### 2.1. Controller Area Network Bus with Flexible Data Rate

Retaining the fundamental structure of the classic CAN bus, the CAN-FD bus introduces pivotal enhancements. During the data transmission phase, it can support speeds up to 5 Mbps or more, significantly surpassing the 1 Mbps limit of the traditional CAN bus. Furthermore, the data frame size has been expanded to accommodate up to 64 bytes of payload, a substantial increase from the 8-byte payload of the classic CAN, thus markedly boosting data transmission efficiency. These improvements in data transmission speed and capacity, devised for modern automotive electronics and high-speed industrial automation systems, cater to more complex and data-intensive applications.

**Figure 2:**
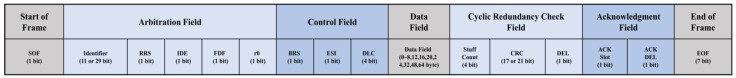
The structure of the CAN-FD frame.

The CAN-FD data frame is illustrated in Figure 2, which includes several critical components [37,38,39]:

- **SOF**: The Start of Frame (SOF) bit is utilized for synchronization and to alert all nodes about the commencement of a CAN-FD message transmission. It is consistently set to 0.
- **Identifier**: Each ECU has a unique identifier code, known as the Identifier (ID), which determines the recipient of a message. The ID spans 11 bits in length, with the message priority dictated by this field; typically, a lower numerical value signifies a higher priority. Consequently, when an ECU faces a choice between two identifiers, the comparison is conducted based on their sequential order. If one identifier exhibits a logical “1” at any position while the other shows a logical “0”, the identifier with the “1” will lose its priority.
- **IDE**: The Identifier Extension (IDE) bit distinguishes between standard and extended frames. A logical “0” indicates an 11-bit ID, whereas a logical “1” indicates a 29-bit ID.
- **DLC**: Data Length Code (DLC) specifies the number of valid bytes within the data field, with the maximum representable byte count being 64.
- **Data Field**: The data field contains the payload data, which is interpreted by the designated receiving ECU.

Our IDS primarily focuses on the frame ID in the arbitration field, the DLC in the control field, the data field, and the timestamp when the frame is recorded. The frame ID, consisting of either 11 or 29 bits, serves to identify messages and facilitate arbitration. Within the CAN-FD protocol, the data field plays a crucial role in the in-vehicle communication network, carrying the actual payload data for communication between various ECUs. This data may encompass information collected from diverse sensors, such as temperature, speed, and acceleration sensors. It controls commands for managing different vehicle components and various status information.

In bus architectures with multiple masters as defined by the CAN-FD protocol, ECUs broadcast messages onto the CAN-FD bus [40]. This architecture employs an arbitration mechanism for message transmission, where the priority of a message is determined by its frame ID; a lower ID value indicates a higher priority frame. This mechanism enables each ECU to attempt bus access and utilization at any time. However, this also introduces vulnerabilities in bus security. The fact that ECU nodes broadcast messages to the CAN-FD bus allows attackers to easily intercept and capture all messages transmitted within the network, potentially leading to Spoofing, Fuzzing, Replay, Scaling, or Ramp attacks; the priority arbitration mechanism may facilitate DoS attacks. Furthermore, automotive companies utilize vehicle communication matrices to define the flow of messages between ECUs, specifying the sending and receiving ECUs, as well as standardizing communication formats, protocols, and parameters. This plays a critical role in ensuring effective arbitration. Without a communication matrix, frame messages often become opaque data, and the configuration methods of communication matrices vary among different companies. Despite this, attackers can still reverse-engineer and modify any frame message within the network without recognizing the physical significance of the data fields, particularly by influencing frame arbitration priorities to execute DoS attacks easily.

### 2.2. Type of Attack

**Figure 3:**
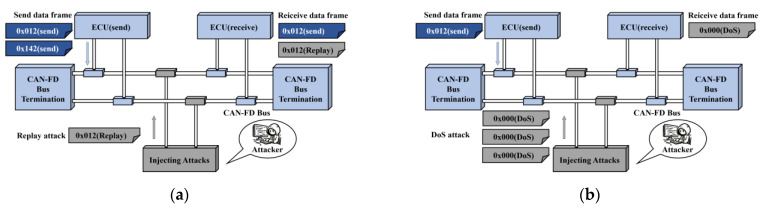

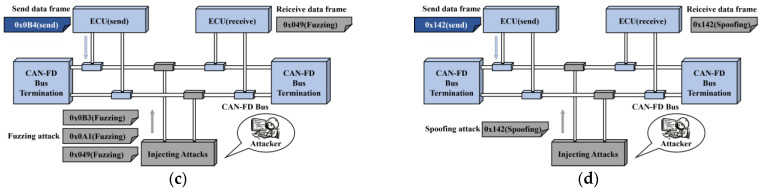
The four types of attacks in this work are (**a**) Replay; (**b**) DoS; (**c**) Fuzzing; and (**d**) Spoofing.

In view of the characteristics described above, any ECU responsible for exchanging information with the external environment could potentially serve as an entry point for attackers to infiltrate the CAN-FD bus system. However, without additional security measures, the frame ID alone cannot verify the identity of the message sender. Such access could lead to various forms of attacks, posing risks to the security of the CAN-FD bus. In this paper, some anomalous attacks examined are shown in Figure 3, including DoS attacks, Fuzzing attacks, Replay attacks, and Spoofing attacks.

**DoS**: Attackers inject high-frequency messages with high-priority frame ID into the network bus, aiming to hinder authorized entities from accessing resources or inducing delays in time-sensitive system operations, thereby disrupting normal communications within the vehicle’s internal network. During an attack, attackers typically send high-priority data or requests to the network, resulting in network congestion that can impact or completely halt data exchanges between ECUs, thereby affecting the normal operation of the vehicle. Particularly in CAN/CAN-FD bus systems, if an attacker sends highest-priority data via an ECU, other ECUs can be prevented from using the bus to transmit messages because of the arbitration mechanism.

**Replay**: Attackers record data from a node over a certain period and replay it later [12]. By duplicating and resending previously transmitted messages in the in-vehicle CAN-FD network, the attacker causes the ECUs to mistakenly respond to outdated signals as if they are current, leading to inappropriate system responses at specific moments. This can lead to repeated or improper actions in the vehicle system, thus disrupting its normal functioning.

**Fuzzing**: A Fuzzing attack involves injecting randomized or semi-randomized data frames into a target’s CAN-FD system, particularly in high-traffic environments. The goal is to test the system’s response to abnormal or invalid inputs [19]. This type of attack, characterized by sending many aberrant or random CAN-FD messages, can result in erratic vehicle behavior like steering wheel jitters, unusual signal light activations, or unexpected gear shifts [15].

**Spoofing**: Attackers transmit entirely forged data frames to a target node, aiming to mimic legitimate data or commands from a real source in the network bus [41]. By simulating authentic ECU communication with these forged CAN-FD messages and altering their control and data fields, attackers deceive other ECUs or the vehicle control system. This can lead to data deviations in ECUs, resulting in loss of vehicle control, execution of unintended operations, or system chaos, ultimately leading to in-vehicle malfunction [42].

**Scaling attack**: A Scaling attack is a cybernetic assault that manipulates the measurement signals to detrimentally affect the performance of a system. In this form of attack, perpetrators alter the genuine measurement signals based on a predetermined scaling factor, which can precipitate outcomes that deviate from the system’s expected responses. For instance, should the speed measurements of an Automated Guided Vehicle (AGV) be artificially inflated, the system might misconstrue the need for increased velocity, consequently initiating inappropriate responses such as excessive acceleration. This could potentially compromise the safety and efficiency of the system. The essence of a Scaling attack lies in its simplicity and the potential chaos it can introduce, necessitating the implementation of robust detection and recovery mechanisms to uphold the stability and security of the system [13,14].

**Ramp attack**: A ramp attack represents a methodical cyber strategy where the attacker incrementally modifies or “ramps” the measurement signals over a period, thereby causing the system’s behavior to stray from its predefined operational conditions. Characterized by a progressive and either ascending or descending alteration of the signals, this attack can surreptitiously steer the system towards suboptimal or even unstable conditions. For example, if an AGV’s navigation system gradually receives increased positional errors, it could lead to the AGV deviating from its planned trajectory, heightening the risk of collisions or inadvertently entering hazardous zones. The covert nature of ramp attacks demands that systems possess highly sensitive anomaly detection capabilities to swiftly identify and counteract these gradual signal changes [13,14].

## 3. Problem Definition and Overview of Method

### 3.1. Problem Definition

This work conceptualizes the anomaly detection of CAN-FD data as a classification task for the target frames. The model considers historical time series data spanning a window of length l, to determine whether the current received data frame is normal or anomalous. Furthermore, it is possible to determine the type of attack causing the anomaly based on the characteristics of the anomalous frames. The concepts of CAN-FD bus anomaly detection are given as follows:

**Definition** **1.** *Anomaly Data Detection. The goal is to differentiate between anomalous and normal messages, effectively executing a binary classification for anomaly detection. To address this challenge, a time-window method is adopted. The message window input for ADDM at the current time *T *is defined as follows:*

(1) $$X_{in,T}=\left[\begin{array}{c} X_{T}\\ X_{T+1}\\ \begin{array}{c} X_{T+2}\\ \vdots \\ X_{T+l-1} \end{array} \end{array}\right]=\left[\begin{array}{ccc} x_{T,1} & x_{T,2} & \begin{array}{cc} \ldots & x_{T,d} \end{array}\\ x_{T+1,1} & x_{T+1,2} & \begin{array}{cc} \cdots & x_{T+1,d} \end{array}\\ \begin{array}{c} x_{T+2,1}\\ \vdots \\ x_{T+l-1,1} \end{array} & \begin{array}{c} x_{T+2,1}\\ \vdots \\ x_{T+l-1,2} \end{array} & \begin{array}{c} \begin{array}{cc} \cdots & x_{T+2,d}\\ \ddots & \vdots \end{array}\\ \begin{array}{cc} \ldots & x_{T+l-1,d} \end{array} \end{array} \end{array}\right],$$

 *where* $X_{t}(t\in \left\{T,\;T+1,T+2,\ldots ,T+l-1\right\})$ *is the extracted features of the whole frame at time* t*,* $x_{t,j}(t\in \left\{T,\;T+1,T+2,\ldots ,T+l-1\right\},j\in \{1,2,3,\ldots ,d\})$ *is the* j*-th feature in the data frame at the time* t*. And the number of features in each frame is* d.

The output of ADDM is a 1-D vector $\hat{Y_{1}}$, which represents the score of result at the time t. A threshold is set as θ. If the $\hat{Y_{1}}<\theta$, the CAN-FD frame will be considered normal; otherwise, an anomaly will be detected.

**Definition** **2.** *Anomaly Classification Detection. Assume the additional goal is to classify all messages into multiple categories, including Benign, Replay, DoS, Fuzzing, Spoofing, Scaling attack, and Ramp attack. This multi-classification task involves not only detecting anomalous patterns but also discerning the specific types of irregularities or malfunctions they may indicate. The complexity of this task lies in differentiating standard operational messages from a spectrum of anomalies, each with its own distinct characteristics and implications for the vehicle’s system integrity.*

For the current time T, let the message window input for the ACDM be denoted as $X_{in,T}$, corresponding to Equation (1). The output of ACDM is then a multi-dimensional vector:

(2) $$\hat{Y_{2}}=\left[p_{Benign},p_{Replay},p_{DoS},p_{Fuzzing},p_{Spoofing},p_{Scaling},p_{Ramp}\right],$$

where $p_{Benign},p_{Replay},p_{DoS},p_{Fuzzing},p_{Spoofing},p_{Scaling},p_{Ramp}$ are used to represent the classification scores of Benign, Replay, DoS, Fuzzing, Spoofing, Scaling attack, and Ramp attack. The number of dimensions is the total number of categories. In our method, this equates to seven, indicating that the model categorizes the input data into one of seven distinct classes.

The model’s output is actually a set of probabilities indicating the likelihood that the input data belongs to each of these classes. Finally, the argmax operation is applied to the output. This operation identifies the index of the maximum value in the output vector, which corresponds to the most likely category that the input data belongs to. The label associated with this index is then taken as the final result of the multi-classification process.

### 3.2. Overview of Methods

**Figure 4:**
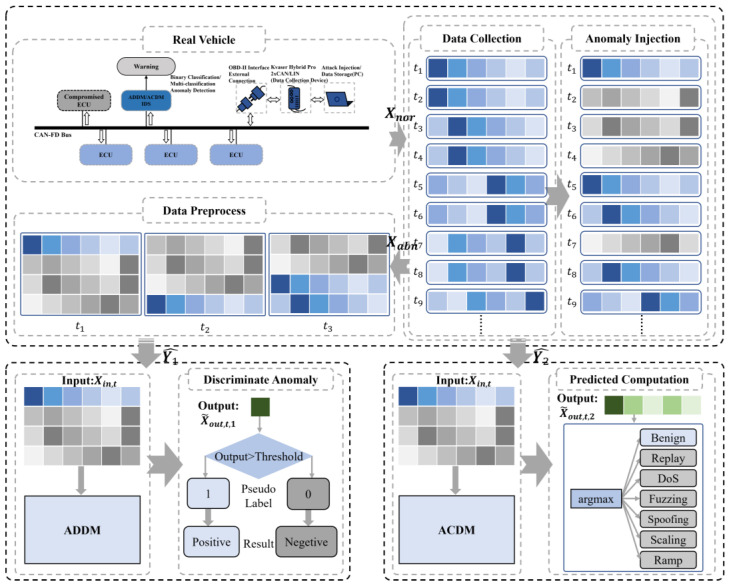
The overview diagram of dual models. The framework is composed of data collection, anomaly injection, data preprocessing, and detection models, including ADDM and ACDM.

As depicted in Figure 4, this work begins with the collection of data from the real vehicle. Subsequently, anomalies are injected into the collected dataset. This is followed by a preprocessing phase. The final step involves using ADDM for binary classification to distinguish between normal and abnormal frames and to identify anomalies. Alternatively, ACDM is used for multiclass classification to facilitate prediction calculations and to ascertain the category of the frames.

## 4. Methodology

### 4.1. Data Collection

We collected a dataset of messages from the CAN-FD bus of a real vehicle. This dataset encompasses CAN-FD messages that are transmitted across the in-vehicle network within a designated timeframe, including the timestamps, CAN-FD ID, data fields, and DLC. Subsequent processing of this dataset is carried out to remove erroneous data, perform numerical conversions, and conduct feature extraction. Following the steps, a refined dataset with normal messages can be obtained, denoted as $X_{nor}$:

(3) $$X_{nor}=\left[\begin{array}{c} X_{1}\\ X_{2}\\ \begin{array}{c} \vdots \\ X_{n} \end{array} \end{array}\right]=\left[\begin{array}{ccc} x_{1,1} & x_{1,2} & \begin{array}{cc} \ldots & x_{1,d} \end{array}\\ x_{2,1} & x_{2,2} & \begin{array}{cc} \cdots & x_{2,d} \end{array}\\ \begin{array}{c} \vdots \\ x_{n,1} \end{array} & \begin{array}{c} \vdots \\ x_{n,2} \end{array} & \begin{array}{c} \begin{array}{cc} \ddots & \vdots \end{array}\\ \begin{array}{cc} \ldots & x_{n,d} \end{array} \end{array} \end{array}\right],$$

where $X_{1},X_{2},\ldots X_{n}$ denote the features in each frame and $x_{t,j}(t\in \left\{1,2,...,n\right\},j\in \left\{1,2,...,d\right\})$ indicates the j-th feature at the time t.

### 4.2. Generation of Anomalous Data

According to the characteristics of CAN-FD communication and attack patterns, we create an attack dataset containing anomalous data, including Replay, DoS, Fuzzing, Spoofing, Scaling, and Ramp attacks, as shown in Algorithm 1.
**Algorithm 1:** Generation of the Attack Dataset**Input:**Dataset without abnormal frame: $X_{nor}$. **Output:**Dataset with abnormal frame: $X_{abn}$. 1:Initial $X_{abn}$ as a copy of $X_{nor}$
2:for i in $N_{DoS}$:3:   $n_{ins}^{DoS}$←A random index number not used before. 4:   for $i^{I}$ in $n_{DoS}$:5:   $x_{DoS}$←DoS frames $x_{DoS}$: set the entire data field to 0.6:   $X_{abn}=X_{abn}\cup x_{DoS}$ ←Insert $x_{DoS}$ into $X_{nor}$ behind row index $n_{ins}^{DoS}$.7:for j in $N_{Fuz}$:8:   $n_{ins}^{Fuz}$←A random index number not used before.9:   for $j^{I}$ in $n_{Fuz}$:10:   $X_{nor}$←Fuzzing frames $x_{Fuz}$: choose integers in ${list}_{Fuz}$ not used as the frame ID and select random value to fill the data field.11:   $X_{abn}=X_{abn}\cup x_{Fuz}$←Insert $x_{Fuz}$ into $X_{abn}$ behind row index $n_{ins}^{Fuz}$
12:for k in $N_{Spo}$:13:   $n_{ins}^{Spo}$←A random index number not used.14:   for $k^{I}$ in $n_{Spo}$:15:   $x_{Spo}$←Spoofing frames $x_{Spo}$: choose integers not used as frame IDs and select substantial values to fill in the data field.16:   $X_{abn}=X_{abn}\cup x_{Spo}$←Insert $x_{Spo}$ into $X_{abn}$ behind row index $n_{ins}^{Spo}$.17:for l in $N_{Rep}$:18:   $n_{ins}^{Rep}$←A random index number not used.19:   for $l^{I}$ in $n_{Rep}$:20:   $x_{Rep}$←Replay frames $x_{Rep}$: repeat the data following the frames.21:   $X_{abn}=X_{abn}\cup x_{Rep}$←Insert $x_{Rep}$ into $X_{abn}$ behind row index $n_{ins}^{Rep}$.22:for h in $N_{Sca}$:23:   $n_{ins}^{Sca}$←A random index number not used.24:   for $h^{I}$ in $n_{Sca}$:25:    $x_{Sca}$←Scaling frames $x_{Sca}$: generate the scaling value data frame 26:   $X_{abn}=X_{abn}\cup x_{Sca}$←Insert $x_{Sca}$ into $X_{abn}$ behind row index $n_{ins}^{Sca}$.27:for g in $N_{Ram}$:28:   $n_{ins}^{Ram}$←A random index number not used.29:   for $g^{I}$ in $n_{Ram}$:30:    $x_{Ram}$←Ramp frames $x_{Ram}$: generate the ramping value data frame. 31:   $X_{abn}=X_{abn}\cup x_{Ram}$←Insert $x_{Ram}$ to $X_{abn}$ behind row index $n_{ins}^{Ram}$.32:return $X_{abn}$


The process of generation involves injecting abnormal data into the normal dataset to simulate attacks, representing the types of anomalous messages an attacker might inject into the CAN-FD bus. In the dataset with abnormal data frames, the numbers of attacks are set as $N_{DoS}$*,* $N_{Fuz}$*,* $N_{Spo}$*,* $N_{Rep}$, $N_{Sca}$*,* $N_{Ram}$, and the number of anomalous frames within each attack are $n_{DoS}$*,* $n_{Fuz}$*,* $n_{Spo}$*,* $n_{Rep}$, $n_{Sca}$*,* $n_{Ram}$, respectively.

The method for generating the attack dataset in this study is outlined in Algorithm 1. N is the length of the initial dataset without any abnormal data frames. The initial step involves determining the number of occurrences for each type of attack and quantifying the anomalous frames involved in each instance. The process begins with generating $X_{abn}$ as a copy of $X_{nor}$ in line 1. From lines 2 to 6, the method for creating DoS anomalous frames is detailed, setting data fields and frame IDs to zero. Lines 7 to 11 outline the generation of Fuzzing anomalous frames, which involves gathering all frame IDs from the original dataset to guarantee their uniqueness, then fabricating abnormal IDs and infusing arbitrary values into the data fields. The construction of Spoofing anomalous frames is elaborated in lines 12 to 16, where previously occurring IDs are used for the frame IDs, while the data fields are populated with highly variable values. Lines 17 to 21 detail the development of Replay anomalies, achieved by replicating successive frames and integrating them into the existing dataset. Lines 22 to 26 describe the creation of the Scaling attack, which is accomplished by applying a scaling factor to the data field values. Lines 27 to 31 detail the generation of the Ramp attack, executed by incrementally adjusting the value in the data field to create a ramp effect. Finally, as shown in lines 32, the dataset is compiled and outputted, encompassing all the injected attacks, thereby completing the attack dataset generation process.

### 4.3. Data Preprocessing

The min-max normalization is applied to the dataset $X_{abn}$, offering several benefits, such as standardizing the range of values and enhancing the algorithm’s performance. For the classification labels assigned to each frame, a binary encoding scheme is used, where “0” indicates the absence and “1” denotes the presence of a particular category. For the binary classification in ADDM, the label can be written as follows:

(4) $$Y_{1}=\left[Y_{Benign}\right],$$

where the value $Y_{Benign}$ denotes the actual label of the CAN-FD frame. And for ACDM, the label can be written as follows:

(5) $$Y_{2}=\left[Y_{Benign},Y_{Replay},Y_{DoS},Y_{Fuzzing},Y_{Spoofing},Y_{Scaling},Y_{Ramp}\right],$$

where the values $Y_{Benign},Y_{Replay},Y_{DoS},Y_{Fuzzing},Y_{Spoofing},Y_{Scaling},Y_{Ramp},$ respectively, correspond to each category. This method facilitates clear differentiation between categories, simplifying the model’s task of classifying each frame. Moreover, it allows for a more precise and efficient analysis of the temporal relationships between consecutive frames, which is crucial for accurate anomaly detection in CAN-FD systems. A set of consecutive CAN-FD bus messages is defined as a single window. The sliding window approach is adopted, selecting a contiguous block of frames as the window. Time windows and strides are used to process the dataset. Then the preprocessed dataset $X_{in,T}$ can be obtained as the input for the model.

### 4.4. ADDM

In the context of anomaly detection in CAN-FD messages, there exists a temporal dependency and numerical relationships that change over time among different moments of CAN-FD messages. LSTM networks can leverage their internal gating mechanisms, including forget gates, input gates, and output gates, to learn the historical patterns of CAN-FD message data, encompassing variations in CAN-FD ID, data fields, and timestamps. Through the influence and retention of state data from historical moments on the current moment’s state data, LSTM networks adapt to changes in data and their dependency patterns across different times. By employing a substantial number of internal parameters for inference, the LSTM layers can find the difference when facing anomalies, which is capable of precisely identifying both long-term and short-term anomalies.

ADDM leverages the strengths of LSTM in processing and memorizing long-term sequential information. Each type of attack, such as DoS and Replay attacks, forms specific sequential patterns within the data stream, which LSTM learns effectively through its gating mechanisms. As network behaviors evolve, so do the characteristics of these attacks. LSTM is capable of continually updating its internal states based on new data, adapting to these changes, and enhancing its ability to detect novel attacks.

**Figure 5:**
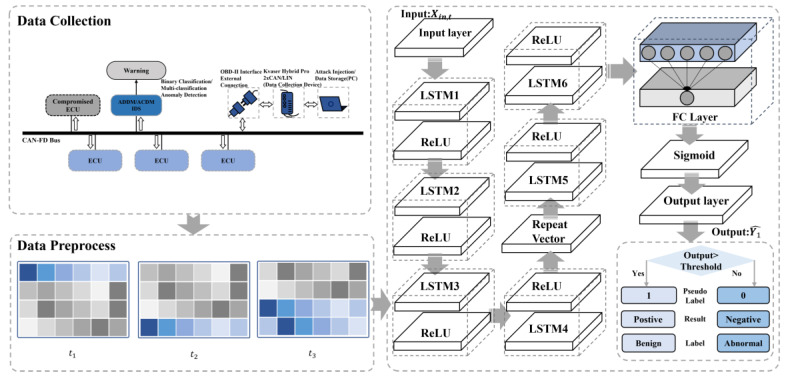
The overview of ADDM architecture.

As shown in Figure 5, this neural network model, ADDM, features a multi-layered architecture designed for sequential data processing, suitable for time series analysis and anomaly detection in data streams. After preprocessing the frame data $X_{abn}$ to be tested, the input $X_{in,T}(X_{in,T}\subseteq X_{abn})$ at time T is fed into the model.

The LSTM unit’s main components include the forget gate, input gate, output gate, and a cell state. The LSTM layers process the input sequence as follows:

(6) $$f_{t}=\sigma (W_{f}*\left[h_{t-1},X_{t}\right]+b_{f}),$$

where $f_{t}$ represents the output of the forget gate, σ is the sigmoid function, constraining the output between 0 and 1, $W_{f}$ is the weight of the forget gate, $b_{f}$ is the bias, $h_{t-1}$ is the previous time step’s hidden state, and $X_{t}$ is one of the time points of $X_{in,T}$.

(7) $$i_{t}=\sigma (W_{i}*\left[h_{t-1},X_{t}\right]+b_{i}),$$

(8) $$\bar{C_{t}}=tanh(W_{c}*\left[h_{t-1},X_{t}\right]+b_{c}),$$

where (7) is the input gate that decides how much new information to add to the cell state. $i_{t}$ is the output of the input gate. $\bar{C_{t}}$ is the new candidate value. $W_{i}$ and $W_{c}$ are the weights for the input gate and candidate value, respectively, and $b_{i}$ and $b_{c}$ are the biases.

(9) $$C_{t}=f_{t}*C_{t-1}+i_{t}*\bar{C_{t}},$$

where the new cell state $C_{t}$ is a combination of two parts: one is the old state scaled by the forget gate, and the other is the new candidate value scaled by the input gate.

(10) $$o_{t}=\sigma (W_{o}*\left[h_{t-1},X_{t}\right]+b_{o}),$$

(11) $$h_{t}=o_{t}*tanh(C_{t}),$$

where the output gate controls the value of the next hidden state. $o_{t}$ is the output of the output gate. $W_{o}$ is the weight for the output gate. $b_{o}$ is the bias. The new hidden state $h_{t}$ is the product of the output gate’s output and the cell state passed through a tanh function. These formulas collectively determine how the LSTM updates its internal state while processing sequential data, allowing it to effectively capture long-term dependencies. The input $X_{in,T}$ at time *T* will be processed sequentially by these formulas. $H_{t}$ denotes the output of the single LSTM layer.

The ADDM network starts with an input layer tailored to ingest the initial data format. The input layer is followed by the LSTM1 layer and the LSTM4 layer. The LSTM layers can capture long-term dependencies and complex patterns in the data.

After the LSTM layers, the last hidden state $H_{t,4}$ including a 1-d vector, is passed to a repeat vector layer. The repeat vector layer serves to bridge the gap between the different LSTM layers. The Repeat Vector layer processes the input sequence as follows:

(12) $$V_{rep,1}=RepeatVector(H_{t,4}).$$

where $V_{rep,1}$ is the matrix that is repeated by $H_{t,4}$.

Through the LSTM5 and LSTM6, the output of the layers can be written as $H_{t,6}$. The subsequent fully connected neural network layer integrates the features extracted by the LSTM layers and applies a linear transformation. There is an activation function after that. In this model, the sigmoid function is chosen to provide a probabilistic output, which is particularly useful for binary classification tasks. $H_{t,6}$ is also the input of the fully connected layer. The fully connected neural network layer is as follows:

(13) $$\hat{Y_{1}}=\sigma_{n1}(W_{n1}*H_{t,6}+b_{n1}).$$

where $\hat{Y_{1}}$ is the output of the model, $\sigma_{n1}$ denotes the sigmoid function, $W_{n1}$ is the weight of the layer, and $b_{n1}$ is the bias.

The network concludes with an output layer that renders the final classification. Binary Cross-Entropy (BCE) is chosen as the loss function, which is employed to quantify the deviation between the predicted probabilities ${\tilde{X}}_{out,t,1}$ and the actual labels. The BCE is shown as follows:

(14) $$BCE=-\frac{1}{N}\sum_{n_{1}=1}^{N}\left[Y_{1}\log\hat{Y_{1}}+\left(1-Y_{1}\right)\log\left(1-\hat{Y_{1}}\right)\right].$$

where N is the total number of samples. The optimization process is guided by the objective of minimizing the error between the predicted outcomes and the actual results.

### 4.5. ACDM

ACDM is designed to classify the frames in the CAN-FD dataset. In this work, the benign frame and several kinds of abnormal frames are all regarded as different types in the classification result.

In anomaly detection of CAN-FD buses, there is often a high dependency in the time series data, and interactions with high correlation are manifested across different moments and IDs in CAN-FD frames. CAN-FD messages at different time points reflect changes in the state of a vehicle at various moments, and the correlations between different data and times are dynamically changing. The attention mechanism is well-suited for calculating the dependencies and correlation relationships between CAN-FD data frames. By utilizing the differences in these correlation attributes, specifically the deviations in attention scores, anomalies deviating from normal patterns can be precisely detected, demonstrating exceptional inferential capabilities.

In ACDM, data at different time points are utilized as Query, Key, and Value in the attention mechanism to calculate the temporal relationships between data points at different times. If a specific frame ID or data pattern is highly correlated with anomalous behavior, the attention mechanism can aid the model by assigning higher weights to these particular time steps, facilitating the recognition of such behavior. ACDM can effectively classify anomalous behaviors: for replay attacks, LSTM can identify repetitive patterns in the time series, while the attention mechanism highlights the time points of these patterns; for DoS attacks, LSTM recognizes short-term substantial increases in priority requests within the data stream, and the attention mechanism helps the model focus on the critical phases at the start of the attack; fuzzing attacks involve attackers sending a large volume of randomly altered packets to the system to trigger errors, with the LSTM learning deviations from normal data and the attention mechanism identifying critical anomaly points; in spoofing attacks, messages mimic other devices or users. Both the LSTM and attention mechanism can recognize these changes through long-term data learning; scaling and ramp attacks gradually impact system performance by scaling or progressively increasing/decreasing certain parameter values. The LSTM can capture such scaling or gradual changes, while the attention mechanism identifies the most significant moments of change.

**Figure 6:**
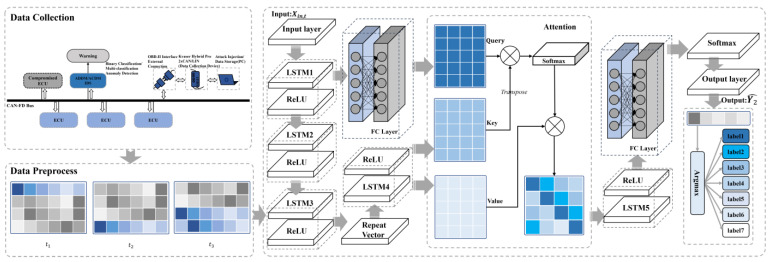
The overview of ACDM architecture.

As depicted in Figure 6, ACDM features a sophisticated architecture that includes stacked LSTM layers augmented by the attention mechanism. The process begins with an input layer that introduces the input sequence $X_{in,t}$ to a series of LSTM layers, specifically LSTM1 to LSTM3. These layers are tasked with capturing the temporal dependencies within the sequence. The outputs of these three LSTM layers are denoted as $H_{t,1}$, $H_{t,2}$, $H_{t,3}$ respectively. Following the LSTM3 layer, a repeat vector layer uses $H_{t,3}$, a 1-d vector, to generate a matrix $V_{rep,2}$ that repeats the information from $H_{t,3}$. The sequence then progresses to the LSTM4 layer, which processes $V_{rep,2}$ to produce the output $H_{t,4}$.

The output $H_{t,1}$ of the LSTM1 layer mentioned in this part is designed to work as a fully connected (FC) layer, as follows:

(15) $$H_{FC}=\sigma_{m1}(W_{m1}*H_{t,1}+b_{m1}).$$

where $\sigma_{m1}$ denotes the sigmoid function, $W_{m1}$ is the weight of the layer, and $b_{m1}$ is the bias. $H_{FC}$ is the output of the FC layer.

Next, the network introduces an attention mechanism module. It employs the output $H_{FC}$ from the FC layer as Query (Q), the output $H_{t,4}$ from the LSTM4 layer as Key (K) and Value (V). The attention mechanism is a crucial component that enables the model to focus on specific parts of the input sequence that are relevant for the classification task. The attention output is generated through a sentence of operations, including dot product, scaling, and applying the Softmax function to generate a weight sum. The sum represents the relative importance of each segment of the input sequence. The process of the attention module is delineated as follows, illustrated from Equations (16)–(19):

(16) $$S_{attn}=Q*K^{T}=H_{FC}*{H_{t,4}}^{T},$$

(17) $$S_{attn,scaled}=\frac{S_{attn}}{\sqrt{d_{K}}},$$

(18) $$\alpha =softmax\left({W_{s}*S}_{attn,scaled}+b_{s}\right),$$

(19) $$A=\alpha *V=\alpha {*H}_{t,4},$$

where Equation (16) computes the attention scores through a dot product between $H_{FC}$ (Q) and ${H_{t,4}}^{T}\;$($K^{T}$). The attention score matrix is $S_{attn}$. In Equation (17), $S_{attn,scaled}$ is scaled $S_{attn}$. $W_{s}$ is the weight. $b_{s}$ is the bias. Equation (18) involves the normalization of these scores using the Softmax function to form the attention weight α. In Equation (19), A represents the output of the attention module.

After passing the attention layer, the data reaches the LSTM5 layer, resulting in an output denoted as $H_{t,5}$. Subsequently, the data are processed through a FC layer, which outputs a 7-d vector, denoted as $Z=\left[z_{1},z_{2},z_{3},z_{4},z_{5},z_{6},z_{7}\right]$. For classification tasks, the final output layer utilizes the Softmax function to calculate the probabilities for each class. The process is detailed as follows:

(20) $$softmax(z_{i})=\frac{e^{z_{i}}}{\sum_{i=1}^{7}e^{z_{i}}},$$

(21) $$\hat{Y_{2}}=\left.softmax\left(\left[z_{1},z_{2},z_{3},z_{4},z_{5},z_{6},z_{7}\right]\right)\right.,$$

where $\hat{Y_{2}}$ is the output of the model.

One-hot coding for the actual labels is used and organized into a one-dimensional vector. To measure the prediction error, the model employs the Categorical Cross-Entropy (CCE) loss for the multi-class classification. The loss function is as follows:

(22) $$CCE=-\sum_{n=1}^{N}\sum_{m=1}^{M}y_{2}log(\hat{y_{2}}),$$

where N is the total number of the samples. $y_{2}$ is the actual class.

## 5. Experiment and Evaluation

### 5.1. Experiment Setup and Evaluation Metric

**Figure 7:**
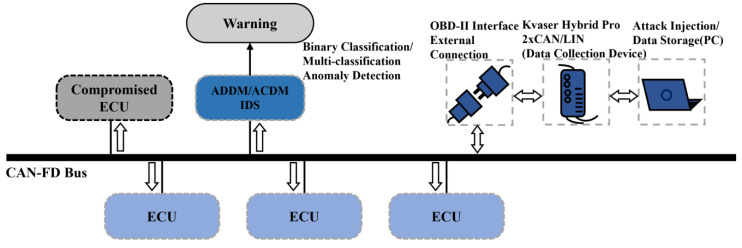
Collection of authentic CAN-FD datasets from a real vehicle.

The two sub-models proposed in this work are validated on two datasets. The first includes original CAN-FD frame data from a real vehicle with L2 autonomous driving capability, using the device shown in Figure 7. The second dataset, known as the CAN-FD Intrusion Dataset, contains Flooding, Fuzzing, and Malfunction attacks and was developed by the Hacking and Countermeasure Research Lab (HCRL) in Korea. Additionally, we adopted the LSTM-based IDS with superior performance proposed by M. D. Hossain et al. [31] and the HyDL-IDS based on CNN-LSTM proposed by Lo et al. [43] for contrast. Our experimental setup is conducted using neural network models developed in the Python-based Keras framework, with computational support from the NVIDIA GEFORCE RTX 4090 GPU. The parameters chosen in the experiment are shown in Table 1.

In this study, the performance of the CAN-FD bus network attack detection method is assessed using several metrics, including accuracy, precision, recall rate, specificity, F1 score [44], and Area Under the Curve (AUC) of the Receiver Operating Characteristic (ROC) curve. These models will be evaluated using these criteria in the subsequent analysis.

Accuracy measures the proportion of true results (both true positives and true negatives) among the total number of cases examined, calculated as $Accuracy=\frac{TP+TN}{TP+TN+FP+FN}$, where TP, TN, FP, and FN represent true positive, true negative, false positive, and false negative, respectively. Precision, or positive predictive value, quantifies the accuracy of positive predictions, defined by the formula $Precision=\frac{TP}{TP+FP}$. Recall rate, also known as sensitivity, measures the ability of a model to identify all relevant instances, with $Recall=\frac{TP}{TP+FN}$. Specificity, indicating the true negative rate, reflects the model’s ability to identify negative results, as given by $Specificity=\frac{TN}{TN+FP}$. Finally, the F1 score is the harmonic mean of precision and recall, offering a balance between the two by computing $F1\;Score=2\times \frac{Precision\times Recall}{Precision+Recall}$, thus capturing the accuracy of the model when dealing with skewed datasets or where false negatives and positives differ crucially.

### 5.2. Experiment with ADDM

**Figure 8:**
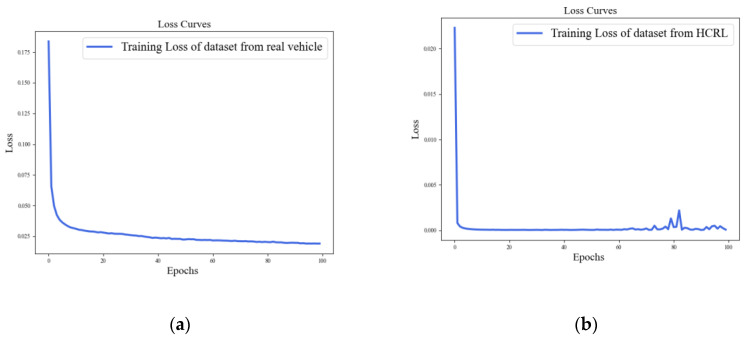
Training loss of the ADDM model on the datasets from a real vehicle and HCRL: (**a**) dataset from an actual real vehicle; (**b**) dataset from HCRL.

**Figure 9:**
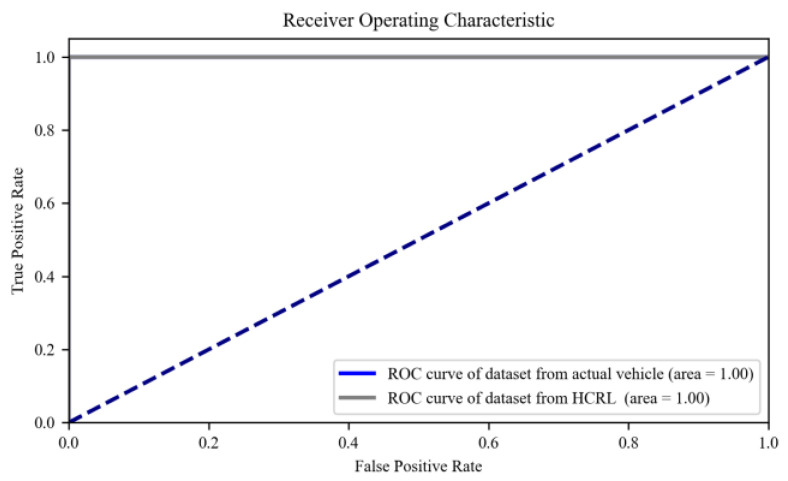
ROC curve of the dataset from the actual vehicle and HCRL.

The loss curves of the ADDM model on both datasets, the actual vehicle and HCRL, are depicted in Figure 8. From the curve, it is observed that the losses in the two models progressively decrease and eventually tend to converge. In Figure 9, the ROC curve for the ADDM model is presented. It is noteworthy that the AUC attains a value of 1 for both datasets, signifying an ideal classification accuracy of all instances. This observation underscores the efficacy of the proposed ADDM model in accurately classifying attacks on the CAN-FD bus network, indicating its robustness in this application domain.

As shown in Table 2, the evaluation metrics of three distinct models, namely ADDM, the LSTM-based model [31], and HyDL-IDS [43], are juxtaposed against two datasets: the Real-vehicle dataset and the HCRL dataset. On the Real-vehicle dataset, the ADDM model demonstrates remarkable performance, achieving an accuracy of 0.9995, a precision of 0.9997, an F1 score of 0.9997, a TNR of 0.9992, and a recall rate of 0.9996. These results notably surpass those of the LSTM-based model, which records an accuracy of 0.9900, a precision of 0.9918, an F1 score of 0.9932, a TNR of 0.9771, and a recall of 0.9946. Furthermore, ADDM’s metrics also exceed those of the HyDL-IDS experiment, which posts an accuracy of 0.9935, a precision of 0.9958, an F1 score of 0.9956, a TNR of 0.9882, and a recall rate of 0.9955. On the HCRL dataset, all three models reach uniformly high scores of 1.0000 or 0.9999 in accuracy, with the ADDM model demonstrating a marginally lower FPR compared with both the LSTM-based model and HyDL-IDS. The results indicate that the accuracy of ADDM on the real-vehicle dataset improves by approximately 0.50% compared with the LSTM-based model and by about 0.41% compared with the HyDL-IDS model.

**Figure 10:**
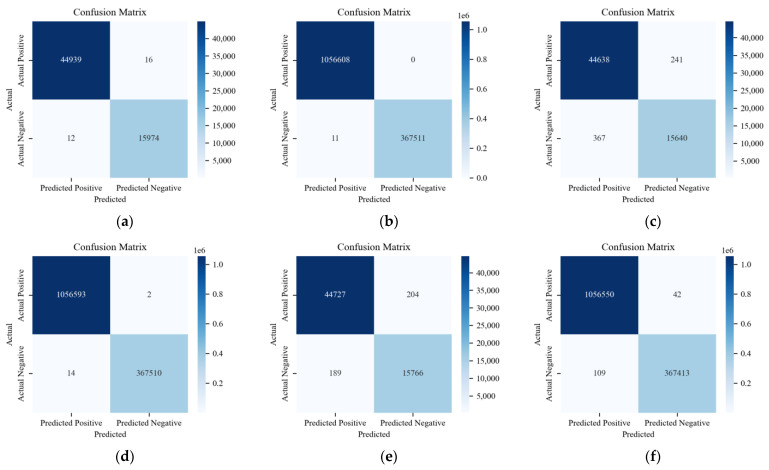
(**a**) Confusion matrix for ADDM trained on the dataset from the actual vehicle; (**b**) confusion matrix for ADDM trained on the dataset from HCRL; (**c**) confusion matrix for LSTM-based model trained on the dataset from the actual vehicle; (**d**) confusion matrix for LSTM-based model trained on the dataset from HCRL; (**e**) confusion matrix for HyDL-IDS trained on the dataset from the actual vehicle; (**f**) confusion matrix for HyDL-IDS trained on the dataset from HCRL.

The confusion matrix reveals specific outcomes for binary classification using the ADDM, the LSTM-based model, and HyDL-IDS. Evidently, in Figure 10, (a) registers a FP count of 12 and a FN count of 16, which is substantially lower than the 367 FPs and 241 FNs depicted in (c) and also lower than the 189 FPs and 204 FNs depicted in (e). When correlating these findings with the parameters described in Table 2, it is apparent that our ADDM model demonstrates superior performance on the Real-vehicle dataset compared with the LSTM-based model and HyDL-IDS. On the HCRL’s dataset, the analysis of classified sample outcomes in (b), (d), and (f) of Figure 10, combined with the data in Table 2, reveals that the ADDM model marginally outperforms the LSTM-based model and HyDL-IDS, recording only eleven FPs and no FN, fewer than the fourteen FPs and two FNs seen in the LSTM-based model and significantly less than the 109 FPs and 42 FNs observed in the result of HyDL-IDS.

The training durations for the ADDM and the two comparative models, including the LSTM-based model and HyDL-IDS, are similar. On the real-vehicle dataset, the inference times for ADDM, the LSTM-based model, and HyDL-IDS are 0.137 ms, 0.105 ms, and 0.119 ms, respectively. On the HCRL dataset, the inference times for ADDM, the LSTM-based model, and HyDL-IDS are 0.102 ms, 0.078 ms, and 0.081 ms, respectively.

### 5.3. Experiment with ACDM

**Figure 11:**
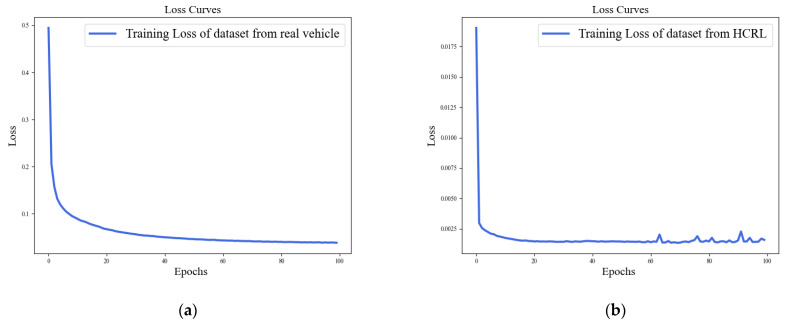
Training loss of the ACDM model on the datasets from a real vehicle and HCRL: (**a**) dataset from an actual real vehicle; (**b**) dataset from HCRL.

The loss curves of the ACDM model on both datasets (actual vehicle and HCRL) are depicted in Figure 11. From the curve, it is observed that the losses in the two models progressively decrease and eventually tend to converge.

Table 3 delineates the performance metrics of the ACDM, LSTM-based model, and HyDL-IDS against various types of network attacks within the two datasets mentioned before. In the real-vehicle dataset, for Replay attacks, the ACDM model achieves detection results of 0.9994, 0.9939, 0.9953, and 0.9996 for accuracy, precision, recall, specificity, and an F1 score of 0.9946, surpassing the performance of an LSTM-based model, which recorded an accuracy of 0.9850, a precision of 0.9447, a recall of 0.7916, and an F1 score of 0.8614, as well as surpassing the HyDL-IDS model, which scored 0.9893 in accuracy, 0.8472 in recall, and 0.9027 in F1 score. For DoS attacks, all three models achieve scores of 1.0000 across all metrics. For Fuzzing attacks, ACDM demonstrated results of 0.9987, 0.9795, 0.9974, 0.9987, and 0.9884 for accuracy, precision, recall, specificity, and F1 score, respectively, exceeding the LSTM-based model’s precision of 0.9570 and accuracy of 0.9966, as well as the HyDL-IDS model’s accuracy of 0.9971 and precision of 0.9614. In the case of Spoofing attacks, ACDM’s performance includes scores of 0.9988, 0.9935, 0.9629, 0.9998, and 0.9780, outperforming the LSTM-based model, which achieved an accuracy of 0.9964 and a recall of 0.9041, and the HyDL-IDS model, which recorded an accuracy of 0.9973 and a recall of 0.9269. For Scaling attacks, ACDM attains metrics of 0.9996, 0.9939, 0.9917, 0.9998, and 0.9928, superior to the LSTM-based model’s accuracy of 0.9991 and recall of 0.9041, and better than the HyDL-IDS results of 0.9991 in accuracy and 0.9824 in precision. For Ramp attacks, ACDM reaches 0.9998, 0.9995, 0.9954, 0.9999, and 0.9975 in accuracy, precision, recall, specificity, and F1 score, surpassing the LSTM-Based model’s recall of 0.9888 and F1 score of 0.9893 and also outperforming the HyDL-IDS model’s recall of 0.9863.

As is shown in Table 3, on the HCRL dataset, for Flooding attacks, all models achieve scores of 1.0000 across all metrics. For Fuzzing attacks, ACDM achieves 0.9999, 0.9999, 1.0000, 0.9999, and 0.9999 in accuracy, precision, recall, specificity, and F1 score, exceeding the LSTM-Based model’s precision of 0.9996 and recall of 0.9997 and surpassing the HyDL-IDS model’s recall of 0.9989. For Malfunction attacks, ACDM achieves perfect scores of 1.0000 across all evaluated metrics, matching the performance of the LSTM-based model, which also attains a score of 1.0000 on every metric. And the performance of ACDM is superior to that of the HyDL-IDS, which scored 0.9999 on all metrics.

On the Real-vehicle dataset, ACDM demonstrates significant performance advantages in handling Replay, DoS, Fuzzing, Spoofing, Scaling, and Ramp attack types. Across crucial performance metrics including accuracy, precision, recall, specificity, and F1 score, the ACDM model surpasses those based on LSTM and HyDL-IDS. Upon evaluation with the HCRL dataset, all models perform well in Flooding and Malfunction attack detection, yet the ACDM maintained a slight edge over the LSTM-based model and HyDL-IDS in Fuzzing attack detection. This comparative analysis reveals the ACDM’s superior detection capabilities, particularly within the Real-vehicle dataset, suggesting its enhanced robustness in identifying nuanced network anomalies.

**Figure 12:**
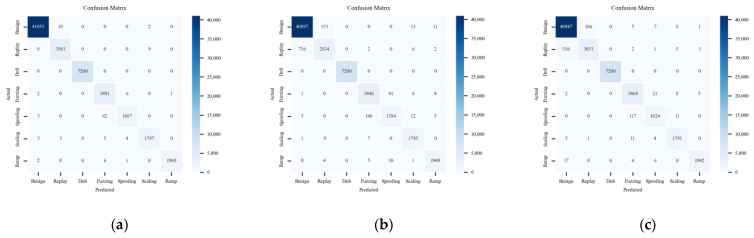
Confusion matrix for the model trained on the dataset from an actual vehicle: (**a**) ACDM; (**b**) LSTM-based model; and (**c**) HyDL-IDS.

Figure 12 displays the result of multi-classification with the labels in subplots (a), (b), and (c), which correspond to different types of CAN-FD frame data. Those express the quantitative relationship between the predicted and actual class labels. It can be seen that the performance of the ACDM model is superior to the LSTM-based model and HyDL-IDS for correctly classified positive samples.

**Figure 13:**
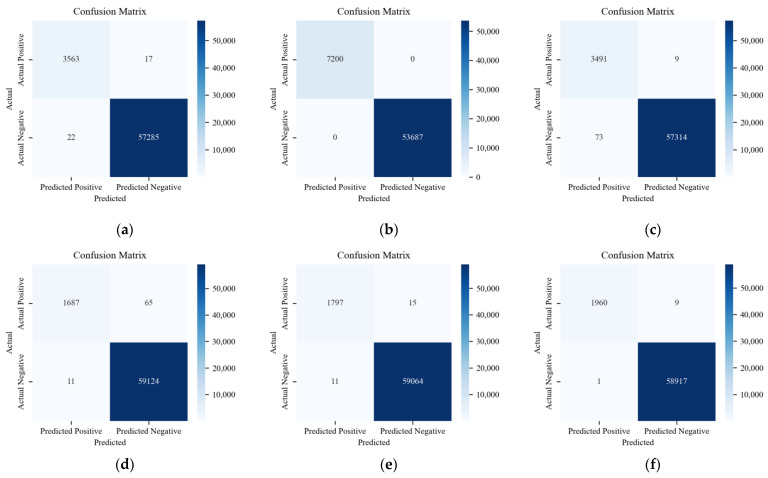
The six figures are the classification results of ACDM trained on the dataset from the actual vehicle: (**a**) Confusion matrix about Replay; (**b**) confusion matrix about Dos; (**c**) confusion matrix about Fuzzing; (**d**) confusion matrix about Spoofing; (**e**) confusion matrix about Scaling attack; and (**f**) confusion matrix about Ramp attack.

**Figure 14:**
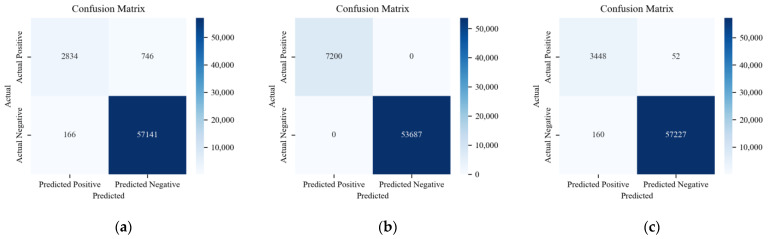

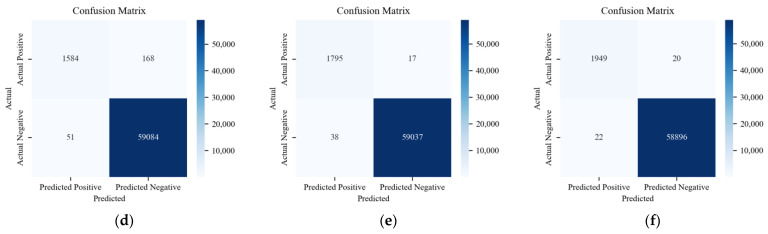
The six figures are the classification results of the LSTM-based model trained on the dataset from the actual vehicle: (**a**) confusion matrix about Replay; (**b**) confusion matrix about DoS; (**c**) confusion matrix about Fuzzing; (**d**) confusion matrix about Spoofing; (**e**) confusion matrix about Scaling attack; and (**f**) confusion matrix about Ramp attack.

**Figure 15:**
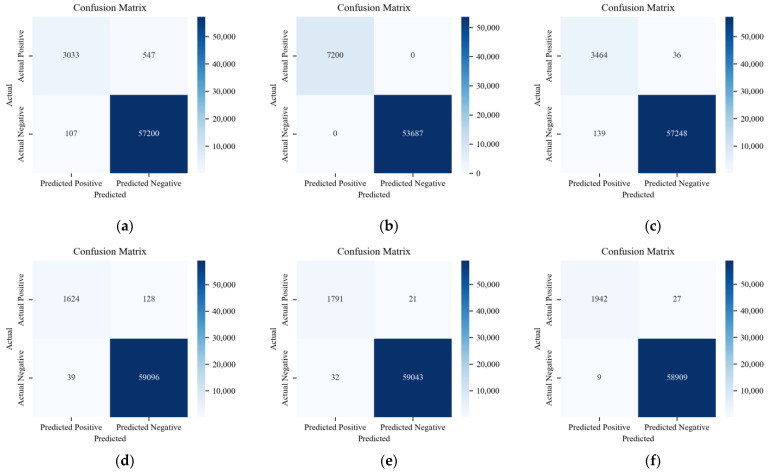
The six figures are the classification results of HyDL-IDS trained on the dataset from the actual vehicle: (**a**) confusion matrix about Replay; (**b**) confusion matrix about DoS; (**c**) confusion matrix about Fuzzing; (**d**) confusion matrix about Spoofing; (**e**) confusion matrix about Scaling attack; and (**f**) confusion matrix about Ramp attack.

Figure 13, Figure 14 and Figure 15 consist of subplots that demonstrate the comparative classification outcomes for three distinct models when applied to a real-vehicle dataset across various attack categories. Specifically, for the Replay attack, subplot (a) in Figure 14 illustrates 746 FNs and 166 FPs, and subplot (a) in Figure 15 shows 547 FNs and 107 FPs. These figures significantly exceed the 17 FNs and 22 FPs recorded by the ACDM model in its respective subplot, highlighting ACDM’s superior classification capability for Replay attacks. In the context of DoS attacks, subplots (b) from Figure 13, Figure 14 and Figure 15 all exhibit 0 FN and FP instances, indicating effective DoS anomaly detection by all models. For Fuzzing attacks, subplots (c) of Figure 14 and Figure 15 reveal a higher occurrence of FNs and FPs in the LSTM-based model, with fifty-two FNs and one hundred and sixty FPs, and in the HyDL-IDS model, with thirty-six FNs and one hundred and thirty-nine FPs, compared with the nine FNs and seventy-three FPs reported in Figure 13’s subplot (c). This underscores the enhanced efficiency of the ACDM model in detecting Fuzzing anomalies. Spoofing anomaly detection, as depicted in subplot (d) of Figure 13, shows fewer FNs and FPs, specifically 65 FNs and 11 FPs, compared with 168 FNs and 51 FPs in Figure 14’s subplot (d) and 128 FNs and 39 FPs in Figure 15’s subplot (d), indicating ACDM’s superior performance in discerning Spoofing anomalies. Regarding Scaling attacks, ACDM’s results include 15 FNs and 11 FPs, which are fewer than the 17 FNs and 38 FPs observed in the LSTM-based model and also fewer than the 21 FNs and 32 FPs in the HyDL-IDS model. For Ramp attacks, ACDM’s outcomes feature nine FNs and one FP, which are less than the twenty FNs and twenty-two FPs in the LSTM-based model and the twenty-seven FNs and nine FPs in the HyDL-IDS model, further illustrating the robustness of ACDM in classifying various cyber threats.

A low recall rate in the LSTM-based model and HyDL-IDS for Replay is attributed to a high quantity of FNs, signifying those numerous actual positive cases remain undetected by the model. For anomaly detection, the comprehension of an event’s context within its sequence is critical. Replay anomalies are characterized by their repetitive nature and thus possess strong contextual correlations, which the LSTM-based model and HyDL-IDS, devoid of the attention mechanism inherent in ACDM, fail to sufficiently capture, resulting in an elevated count of missed positive samples. The distinct characteristics of DoS anomalies, marked by their high-priority features, are accurately identified by all the models without any false positives. When addressing Fuzzing and Spoofing anomalies, the ACDM model’s enhanced feature learning and contextual understanding surpass the LSTM-based model, leading to the superior performance of ACDM. Conclusively, the ACDM demonstrates better detection effectiveness on the real-vehicle dataset and has a broader applicability when compared with the LSTM-based model and HyDL-IDS.

**Figure 16:**
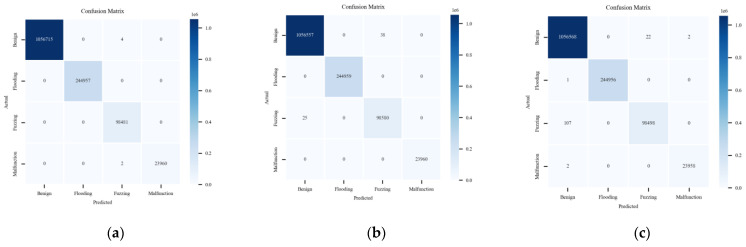
Confusion matrix for the model trained on the dataset from HCRL: (**a**) ACDM; (**b**) LSTM-based model; and (**c**) HyDL-IDS.

In addition to the previous analysis, three methods are compared on the CAN-FD Intrusion Dataset from HCRL. As shown in the multi-class results of Figure 16, data are presented for four labels: Benign, Flooding, Fuzzing, and Malfunction.

**Figure 17:**
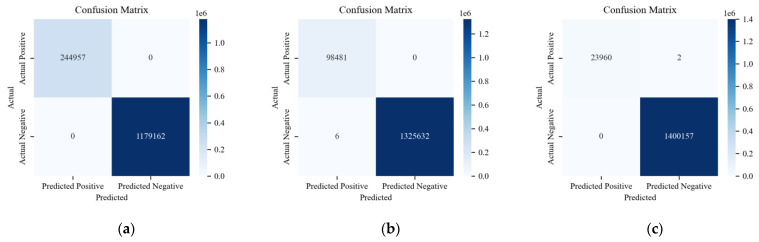
The classification results of ACDM trained on the dataset from HCRL: (**a**) confusion matrix about Flooding; (**b**) confusion matrix about Fuzzing; and (**c**) confusion matrix about Malfunction.

**Figure 18:**
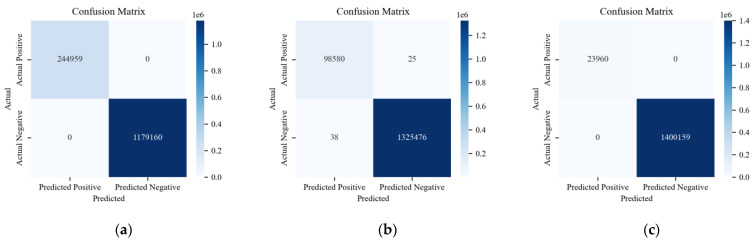
The classification results of the LSTM-based model trained on the dataset from HCRL: (**a**) confusion matrix about Flooding; (**b**) confusion matrix about Fuzzing; and (**c**) confusion matrix about Malfunction.

**Figure 19:**
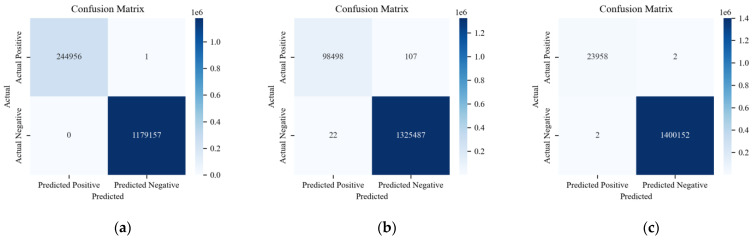
The classification results of HyDL-IDS trained on the dataset from HCRL: (**a**) confusion matrix about Flooding; (**b**) confusion matrix about Fuzzing; and (**c**) confusion matrix about Malfunction.

Figure 17, Figure 18 and Figure 19 show the detection results of each type of attack message in multi-class anomaly monitoring. Comparing each subplot and the data in Table 3, for Flooding attacks, both ACDM and the LSTM-based model have no FNs or FPs, whereas HyDL-IDS has one FN. For Fuzzing attacks, ACDM results show zero FNs and six FPs, fewer than the LSTM-based model, which records 25 FNs and 38 FPs, and significantly less than HyDL-IDS, which detects 107 FNs and 22 FPs. For Malfunction attacks, ACDM’s results include two FNs and no FPs, aligning with the detection performance of the LSTM-based model and HyDL-IDS. This indicates that our model has achieved better performance than that of the LSTM-based model and HyDL-IDS in detecting and classifying anomalies in large-scale datasets containing abnormalities including Flooding, Fuzzing, and Malfunction.

The integration of LSTM with attention mechanisms, as compared with using LSTM alone and HyDL-IDS, offers significant advantages in the context of multi-classification anomaly detection problems. Firstly, the attention mechanism enhances the model’s ability to focus on specific parts of the input sequence that are more relevant for making a decision. This is particularly useful in anomaly detection, where certain patterns or anomalies might be subtle and not uniformly distributed throughout the data. Secondly, the combination allows the model to capture long-term dependencies more effectively. While LSTM units are adept at handling such dependencies, the attention layer provides an additional filter to emphasize the most critical information over longer sequences, which can be crucial in identifying complex or temporally distant anomalies. Thirdly, the LSTM with attention mechanism can improve interpretability. At last, this approach can lead to improvements in accuracy and efficiency. By directing the model’s focus to key areas, the attention mechanism can reduce the influence of irrelevant data, potentially leading to better performance in detecting anomalies.

The training times for the ACDM and the two comparative models, including the LSTM-based model and HyDL-IDS, are closely aligned. On the real-vehicle dataset, the inference times for ACDM, the LSTM-based model, and HyDL-IDS are recorded at 0.141 ms, 0.099 ms, and 0.123 ms, respectively. On the HCRL dataset, the inference times for ADDM, the LSTM-Based model, and HyDL-IDS are 0.126 ms, 0.070 ms, and 0.093 ms, respectively.

### 5.4. Disscusion

In the detection of anomalies in CAN-FD message frames, models based on attention and LSTM offer unique advantages compared with those solely based on LSTM or the CNN-LSTM-based model HyDL-IDS. Firstly, the attention mechanism allows the model to more effectively focus on key features within anomalous behaviors because it dynamically adjusts the model’s focus on different parts of the input data, thereby enhancing the model’s precision in recognizing abnormal patterns. In contrast, traditional LSTM models, while capable of capturing long-term dependencies in time-series data, may lack sufficient flexibility when faced with complex or subtle anomalies. The CNN-LSTM-based model HyDL-IDS, although it strengthens feature extraction through convolutional layers, does not match the dynamic adjustment capabilities of attention-based models in terms of temporal correlations within the sequence. Thus, models combining attention and LSTM not only retain the advantages of LSTM in processing time-series data but also enhance the ability to identify critical features in abnormal signals through the attention mechanism, providing more accurate and flexible anomaly detection performance.

Compared with the baseline methods, the approach proposed in this paper conducts refined multi-class anomaly detection that includes six types of attacks rather than the inference for individual attack types as done by the comparative methods. By incorporating the attention mechanism, the detection accuracy is successfully improved for Replay attacks, significantly reducing FNs and FPs. The approach proposed in this paper achieves ideal results for DoS attacks, comparable to the comparative methods. For Fuzzing, Spoofing, Scaling, and Ramp attacks, our method improves detection performance by combining the attention mechanism with LSTM and stacking LSTM layers. Besides using a real-vehicle dataset that we collected and injected with attacks, the model proposed in this work is also validated on the HCRL-provided open-source CAN-FD Intrusion Dataset, where results show that our proposed method outperforms the comparative methods.

## 6. Conclusions

This study proposes two anomaly detection models based on LSTM and temporal attention mechanisms for the CAN-FD bus, focusing on the temporal characteristics between the CAN-FD frames and aiming to identify the anomalous data and type of the attack. The first sub-model, ADDM, utilizes stacked LSTM to perform binary classification of normal and anomalous data within the CAN-FD dataset, thereby detecting external attacks. The second sub-model, ACDM, combines LSTM with an attention mechanism and capitalizes on the characteristics of various anomalous messages to classify messages within the CAN-FD dataset, thus identifying the types of external attacks. Compared with existing advanced LSTM-based and HyDL-IDS, our method exhibits superior performance in detection, achieving an improvement in both the real-vehicle dataset and the CAN-FD Intrusion Dataset from HCRL. For binary normal/abnormal anomaly detection on the real-vehicle dataset, ADDM achieves a classification accuracy that is 0.50% higher than the LSTM-based model and 0.41% higher than HyDL-IDS; on the HCRL dataset, the classification accuracy of our ADDM matches that of the LSTM-based model and HyDL-IDS, all at 99.99%. For the refined six-category multiclass anomaly detection on the real-vehicle dataset, ACDM achieved a maximum accuracy improvement of 1.44% compared with the LSTM-based model and 1.01% compared with HyDL-IDS. On the HCRL dataset, the multiclass detection average accuracy of ACDM is on par with the LSTM-based model, both at 100%, and improved by 0.01% compared with the accuracy of HyDL-IDS. And fewer FNs and FPs demonstrate the broader applicability of ACDM. Based on statistics on automotive attack vectors over the past decade, attacks directly or indirectly through the CAN/CAN-FD bus account for approximately 13.61% of all external attacks. This study can mitigate about 9% of them.

In the future, in order to delve deeper into anomaly detection and its countermeasures regarding the physical significance of anomalous signals, we aim to develop a more adaptive framework capable of dynamically adjusting to new types of network threats. This will allow us to maintain robust defense mechanisms as automotive technologies continue to evolve. To achieve these objectives, we will use the causal relationships of CAN/CAN-FD messages as inputs and learn the structural and positional relationships between graph nodes through graph structures. By utilizing transformers, we aim to carry out predictions, classifications, or scoring mechanisms to fulfill the purposes of anomaly detection and defense.

## Figures and Tables

**Table 1:** The configuration of model parameters.

Parameters	Value	
	ADDM	ACDM
Learning Sate	$10^{-3}$	$10^{-4}$
Epochs	100	100
Batch Size	128	128
LSTM Unit Number	256,256,64,64,32	512,512,128,128,64
Dropout	0.2	0.2
Window Size	4	4
Stride	1	1
Optimizer	Adam	Adam

**Table 2:** Table for results and evaluation metrics of ADDM, LSTM-based models, and HyDL-IDS on the different datasets.

	Type of Attack	TPR (Recall)	FPR	TNR (Specificity)	FNR	Accuracy	Precision	F1 Score
Real-vehicle dataset	ADDM	**0.9996**	**0.0008**	**0.9992**	**0.0004**	**0.9995**	**0.9997**	**0.9997**
	LSTM-based model	0.9946	0.0229	0.9771	0.0054	0.9900	0.9918	0.9932
	HyDL-IDS	0.9955	0.0118	0.9882	0.0045	0.9935	0.9958	0.9956
HCRL dataset	ADDM	**1.000**	**3 × 10^−5^**	**0.9999**	**0**	**0.9999**	**0.9999**	**0.9999**
	LSTM-based model	0.9999	4 × $10^{-5}$	**0.9999**	2 × $10^{-6}$	**0.9999**	**0.9999**	**0.9999**
	HyDL-IDS	0.9999	0.0003	0.9997	4 × $10^{-5}$	**0.9999**	**0.9999**	**0.9999**

**Table 3:** Evaluation metrics of ACDM, LSTM-based models, and HyDL-IDS on the different datasets.

Dataset	Type of Attack	Model	Accuracy	Precision	Recall	Specificity	F1 Score
Real-vehicle dataset	Replay	ACDM	**0.9994**	**0.9939**	**0.9953**	**0.9996**	**0.9946**
		LSTM-based model	0.9850	0.9447	0.7916	0.9971	0.8614
		HyDL-IDS	0.9893	0.9659	0.8472	0.9981	0.9027
	DoS	ACDM	**1.0000**	**1.0000**	**1.0000**	**1.0000**	**1.0000**
		LSTM-based model	**1.0000**	**1.0000**	**1.0000**	**1.0000**	**1.0000**
		HyDL-IDS	**1.0000**	**1.0000**	**1.0000**	**1.0000**	**1.0000**
	Fuzzing	ACDM	**0.9987**	**0.9795**	**0.9974**	**0.9987**	**0.9884**
		LSTM-based model	0.9966	0.9570	0.9851	0.9973	0.9709
		HyDL-IDS	0.9971	0.9614	0.9897	0.9976	0.9754
	Spoofing	ACDM	**0.9988**	**0.9935**	**0.9629**	**0.9998**	**0.9780**
		LSTM-based model	0.9964	0.9688	0.9041	0.9991	0.9353
		HyDL-IDS	0.9973	0.9765	0.9269	0.9993	0.9511
	Scaling	ACDM	**0.9996**	**0.9939**	**0.9917**	**0.9998**	**0.9928**
		LSTM-based model	0.9991	0.9793	0.9906	0.9994	0.9849
		HyDL-IDS	0.9991	0.9824	0.9884	0.9995	0.9854
	Ramp	ACDM	**0.9998**	**0.9995**	**0.9954**	**0.9999**	**0.9975**
		LSTM-based model	0.9993	0.9888	0.9898	0.9996	0.9893
		HyDL-IDS	0.9994	0.9954	0.9863	0.9998	0.9908
HCRL dataset	Flooding	ACDM	**1.0000**	**1.0000**	**1.0000**	**1.0000**	**1.0000**
		LSTM-based model	**1.0000**	**1.0000**	**1.0000**	**1.0000**	**1.0000**
		HyDL-IDS	**1.0000**	**1.0000**	**1.0000**	**1.0000**	**1.0000**
	Fuzzing	ACDM	**0.9999**	**0.9999**	**1.0000**	**0.9999**	**0.9999**
		LSTM-based model	**0.9999**	0.9996	0.9997	**0.9999**	0.9997
		HyDL-IDS	**0.9999**	0.9998	0.9989	**0.9999**	0.9993
	Malfunction	ACDM	**1.0000**	**1.0000**	**1.0000**	**1.0000**	**1.0000**
		LSTM-based model	**1.0000**	**1.0000**	**1.0000**	**1.0000**	**1.0000**
		HyDL-IDS	0.9999	0.9999	0.9999	0.9999	0.9999

## Data Availability

The data presented in this study are available on request from the corresponding author.
